# *Panax ginseng* improves physical recovery and energy utilization on chronic fatigue in rats through the PI3K/AKT/mTOR signalling pathway

**DOI:** 10.1080/13880209.2023.2169719

**Published:** 2023-01-25

**Authors:** Guolei Zhang, BoFan Lu, Enhui Wang, Wei Wang, Zheng Li, Lili Jiao, Hui Li, Wei Wu

**Affiliations:** Jilin Ginseng Academy, Changchun University of Chinese Medicine, Changchun, China

**Keywords:** Herb, antifatigue, UPLC–MS, saponin

## Abstract

**Context:**

*Panax ginseng* C. A. Meyer (Araliaceae) is a tonic herb used in ancient Asia.

**Objective:**

This study investigated the antifatigue effect of *P. ginseng* on chronic fatigue rats.

**Materials and methods:**

Sprague-Dawley rats were divided into control, model and EEP (ethanol extraction of *P. ginseng* roots) (50, 100 and 200 mg/kg) groups (*n* = 8). The rats were subcutaneously handled with loaded swimming once daily for 26 days, except for the control group. The animals were intragastrically treated with EEP from the 15th day. On day 30, serum, liver and muscles were collected, and the PI3K/Akt/mTOR signalling pathway was evaluated.

**Results:**

The swimming times to exhaust of the rats with EEP were significantly longer than that without it. EEP spared the amount of muscle glycogen, hepatic glycogen and blood sugar under the chronic state. In addition, EEP significantly (*p* < 0.05) decreased serum triglycerides (1.24 ± 0.17, 1.29 ± 0.04 and 1.20 ± 0.21 vs. 1.58 ± 0.13 mmol/L) and total cholesterol (1.64 ± 0.36, 1.70 ± 0.15 and 1.41 ± 0.19 vs. 2.22 ± 0.19 mmol/L) compared to the model group. Regarding the regulation of energy, EEP had a positive impact on promoting ATPase activities and relative protein expression of the PI3K/Akt/mTOR pathway.

**Conclusions:**

Our results suggested that EEP effectively relieved chronic fatigue, providing evidence that *P. ginseng* could be a potential dietary supplement to accelerate recovery from fatigue.

## Introduction

Fatigue is a subjective discomfort, defined as a lack of energy, diminished endurance and prolonged recovery after physical activity, making it impossible to complete routine activities or work (Zhang et al. 2020). Chronic or cumulative fatigue can seriously affect an individual’s performance and significantly reduce their quality of life. Many studies have shown that fatigue is associated with disturbances in energy metabolism (Anand et al. 2012). Some Chinese herbal medicines have positive antifatigue effects, in which *Panax ginseng* C. A. Meyer (Araliaceae) is a representative one.

As the king of the herbs, *P. ginseng* has been used as both nutrition and medicine for many years (Lu et al. 2021). Saponins are the main components of *P. ginseng* (Bao et al. 2016), which could increase muscle mass, improve exercise performance, optimize energy utilization and decrease fatigue-related metabolites (Arring et al. 2018; Kim et al. 2020; Nurpadila et al. 2021). An understanding of the antifatigue effects of saponins has increased with the development of modern cell biology, molecular biology and other disciplines: Zhuang et al. (2014) identified the antifatigue effects of ginsenoside Rb1 on aged small intestine resected rats related to the up-regulated enzymes of Nrf2; Yang QY et al. (2018) pointed out that the effect of Rg3 increased the journey distance and rearing frequency of mice by activating SIRT1 and suppressing p53. However, the above reports were all on acute fatigue, and there is insufficient research on the improving effect of *P. ginseng* on chronic fatigue.

Fatigue is closely related to the disequilibrium of the energy supply/depletion resulting in the production of an insufficient amount of energy in active muscles or the depletion of endogenous substrates such as carbohydrates (Chuckravanen et al. 2019). The resulting changes in exercise performance occur through the integration of multiple signalling pathways that can control and modulate the transcription factors and nutrient synthesis and degradation (Morita et al. 2013; Correia et al. 2020). Therefore, the recovery process from exercise-induced fatigue must repair the damage that already occurred in the body and/or promote the elimination of metabolites accumulated during the exercise. Furthermore, accumulating evidence indicates that the mammalian target of rapamycin (mTOR), as a nutrient sensor and a typical serine/threonine protein kinase, is a very important regulator of energy metabolism.

The mTOR signalling cascade aggregates and integrates signalling factors that play a significant role in sugar, lipid and protein metabolism (Szwed et al. 2021; Amorim et al. 2022). In the present study, a chronic fatigue model of rats was established through prolonged swimming training, and the antifatigue effect of *P. ginseng* was evaluated in terms of sugar, fat and protein metabolism and the key targeted signalling pathway PI3K/Akt/mTOR to provide a new perspective on the pathogenesis of chronic fatigue and the therapeutic mechanism of *P. ginseng*.

## Materials and methods

### Materials

*P. ginseng* roots were purchased from Hongjiu Biological Technology Co., Ltd. (Jilin, China) (batch number: JLPG18-1) and authenticated by Dr. Bo Li. The sample was stored in a glass jar in Jilin Ginseng Academy, Jilin Province, Changchun, China.

The assay kit of blood glucose (B-Gy, lot no. F006-1-1), hepatic/muscle glycogen (H-Gn lot no. A043-1-1, M-Gn lot no. A043-1-1), blood urea nitrogen (BUN, lot no. C013-2-1), free fatty acids (FFA, lot no. A042-1-1), lactic acid (LD, lot no. A019-2-1), triglycerides (TGs, lot no. A110-1-1) and total cholesterol (TC, lot no. A111-1-1) were purchased from Nanjing Jiancheng Bioengineering Institute (Nanjing, China). In addition, biological samples were stored in the PHCBI cryogenic medical refrigerator, and preserved at −80 °C (Panasonic, Tokyo, Japan).

### Preparation of EEP

The ethanol extract of *P. ginseng* (EEP) root was obtained with the ethanol reflux extraction method, followed by microporous adsorption resin. Briefly, *P. ginseng* (500 g) was refluxed three times with 5000 mL of ethanol with a concentration of 70% (v:v). Then, the extract was concentrated under vacuum rotary evaporation. Next, the extracts were diluted and loaded onto D101 microporous resins. Finally, the 70% ethanol eluate was collected, concentrated and lyophilized (Tokyo Rikakikai, Bunkyo, Japan).

### Liquid chromatography–mass spectrometry and UV for saponin analysis

EEP was further analysed for monomer composition with ultra-performance liquid chromatography–tandem mass spectrometry (UPLC–MS/MS) using Thermo TSQ Endura 6520 (Thermo Fisher, Waltham, MA). At the same time, EEP were further detected using a reverse-phase C_18_ column (100 mm × 2.1 mm) with a particle size of 2.6 µm (Thermo Fisher, Waltham, MA) at 35 °C with the ESI ion source. In addition, the mobile phase, composed of water with 0.1% formic acid (A) and acetonitrile (B) gradient, was filtered before use. Furthermore, the flow rate was set to 200 µL/min with an injected volume of 2 µL, while ion source conditions in the mass spectrometer were set as follows: capillary temperature = 350 °C; S-lens RF level = 65.0; spray voltage = 3.5 kV; sheath gas (N_2_) flow rate = 50 arbitrary units; auxiliary gas (N_2_) flow rate = 5; the scan range was 100–1300 *m/z*. Moreover, the gradient of the mobile phase A:B (85:15, v:v) was maintained for 20 min, then adjusted to (10:90, v:v) at *t* = 75 min and maintained for *t* = 80 min, followed by a linear to (85:15, v:v) at *t* = 80.5 min and maintained for *t* = 90 min for post-run. In addition, monomers were identified in both positive and negative modes, followed by the product scan.

EEP saponin content was detected with the colorimetric method. Briefly, 100 µL aliquots were dried in a water bath (60 °C). Then, the residue was dissolved and mixed with vanillin/glacial acetic acid (5%). The mixture was incubated at 60 °C for 15 min, and the reaction was terminated by 5 mL of glacial acetic acid. Moreover, the absorbance was measured using a UV spectrophotometer at 550 nm, while a standard solution (1 mg/mL) was used for content calculation.

### Animals

Male Sprague-Dawley (SD) rats (6–8 weeks) weighing 200–220 g were purchased from Changchun Yisi Laboratory Animal Centre (SCXK (JI) 2019-0007) (Changchun, China). Then, six rats were housed in each cage and screened with swimming without loading. The screening method was to record the swimming time of each rat excluding the rats whose average swimming time was less than 50% of the average swimming time within three days. Afterwards, the screened rats were housed on a 12 h light/dark cycle at a controlled temperature and humidity with unlimited access to food and water. Standard food and water were provided *ad libitum*. The protocols for experimental operations and animal treatment were approved by the Institutional Animal Care and Use Committee at Changchun University of Chinese Medicine, Jilin, China (approval number: 20190200).

### Modelling, grouping and administration

In this study, the chronically fatigued model was established using weight-loaded swimming. The rats were placed in a 120 × 80 × 100 cm iron tank (25 ± 1 °C), and 10% of their body weight was tied to the base of the rat’s tail. During swimming training, the rats were forced to keep their limbs keep paddling. Failure to surface within 10 s was defined as exhaustion, and the time to exhaustion was recorded for each rat.

**Figure 1.**
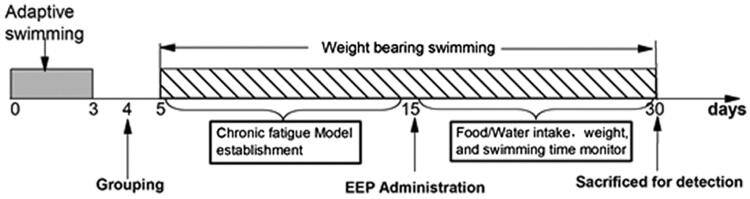
Experiment schedule.

Rats were divided into five groups: the control (control), model (model) and three EEP administration groups, namely low-dose group (EEP-LG, 50 mg/kg), medium-dose group (EEP-MG, 100 mg/kg) and high-dose group (EEP-HG, 200 mg/kg) (*n* = 8). The doses were set according to the which recommended dosage of 3–9 g/day for *P. ginseng*. Afterward, the rats were forced to swim from the 5th to the 30th day to induce and maintain chronic fatigue, except for the control group. Rats were given EEP by intragastric administration from the 15th day, the control and model group were given equal volume of saline. The body weight, food consumption and water intake were measured every two days throughout the experiment. Figure 1 shows the experimental schedule.

### Measurement of biochemical indicators

Five minutes after the last swimming, the rats were anesthetized with ether, and blood samples were collected from the eyeballs. Then, serum was obtained by centrifugation at 1500 rpm for 15 min with Eppendorf Centrifuge 5810 (Eppendorf, Hamburg, Germany), followed by storage at −80 °C for B-Gy, LD, TG, TC, FFA and BUN detection. Afterwards, the rats were sacrificed by cervical dislocation. Next, the liver and soleus muscle samples were collected and snap-frozen in liquid nitrogen, before storage at −80 °C for H-Gn and M-Gn detection. All the biochemical indicators were detected by commercially available kits according to the manufacturer’s protocols (Jiancheng Bioengineering Institute, Nanjing, China).

### Western blot analysis

The soleus muscle tissue was homogenized in an ice-cold RIPA lysis buffer-added protease inhibitor that was an EDTA-free cocktail and phosphate inhibitors (Solarbio, Beijing, China). Protein samples were mixed with the sodium SDS gel loading buffer and denatured and boiled after concentration detection.

Then, the separated proteins were transferred to polyvinylidene fluoride (PVDF) membranes, blocked with TBST for 2 h, and incubated with antibodies (PI3K (p58), anti-Akt/pAkt (ser 473), anti-mTOR/pmTOR (ser 2448), anti-β-actin (Cell Signaling Technology, Boston, MA)) primary overnight at 4 °C. In addition, the membranes were incubated with the horseradish peroxidase-conjugated secondary antibody at 37 °C for 1 h and then washed and detected with an enhanced chemiluminescence (ECL) kit. Furthermore, protein bands adsorbed to membranes were visualized using a Clinx Science Instruments Imaging System (Clinx, Shanghai, China), while grey analysis of Western blot bands was conducted using image-processing software (Image J, National Institutes of Health, Bethesda, MD).

### Statistical analysis

All the statistical analyses were performed using the statistical software SPSS version 19.0 (SPSS, Chicago, IL). The data were presented as mean ± standard deviation (SD). In addition, the parameters were compared using one-way ANOVA and Tukey’s *post hoc* tests. Differences between the groups were significant at *p* value <0.05.

## Results

### Analysis of saponin in extracts

Component analysis of EEP was carried out from two aspects. The total saponin in EEP was determined with Re as the reference; then the saponin were further analysed with mass spectrometry. Taking ginsenoside-Re as the reference, the regression equation of the curve was *Y* = 0.276*X* + 0.0183 with *R*^2^=0.9904. Saponin content in EEP was 78.81%, indicating that after ethanol extraction and macroporous resin treatment, the main component in EEP was saponin.

Furthermore, monomers in EEP were analysed with TSQ-Endura. The identification method was similar to the one described previously (Li H et al. 2019). The molecular weight of 100–1300 Da was analysed in both positive and negative modes. In the positive detection mode, saponin monomers displayed [M + H] ^+^ and [M + Na] ^+^ peaks, while in the negative mode, they exhibited [M–H]^–^ and [M + HCOO]^–^ peaks. In addition, the parent skeleton mainly had two types of glycol linked with different numbers and types of sugars. The sugars attached to the parent skeleton were arabinose (150 Da), rhamnose (164 Da), xylose (150 Da) and glucose (180 Da). Moreover, main chromatographic peaks obtained from the total ion chromatograms were analysed with LC–MS/MS (Wu et al. 2012).

**Figure 2.**
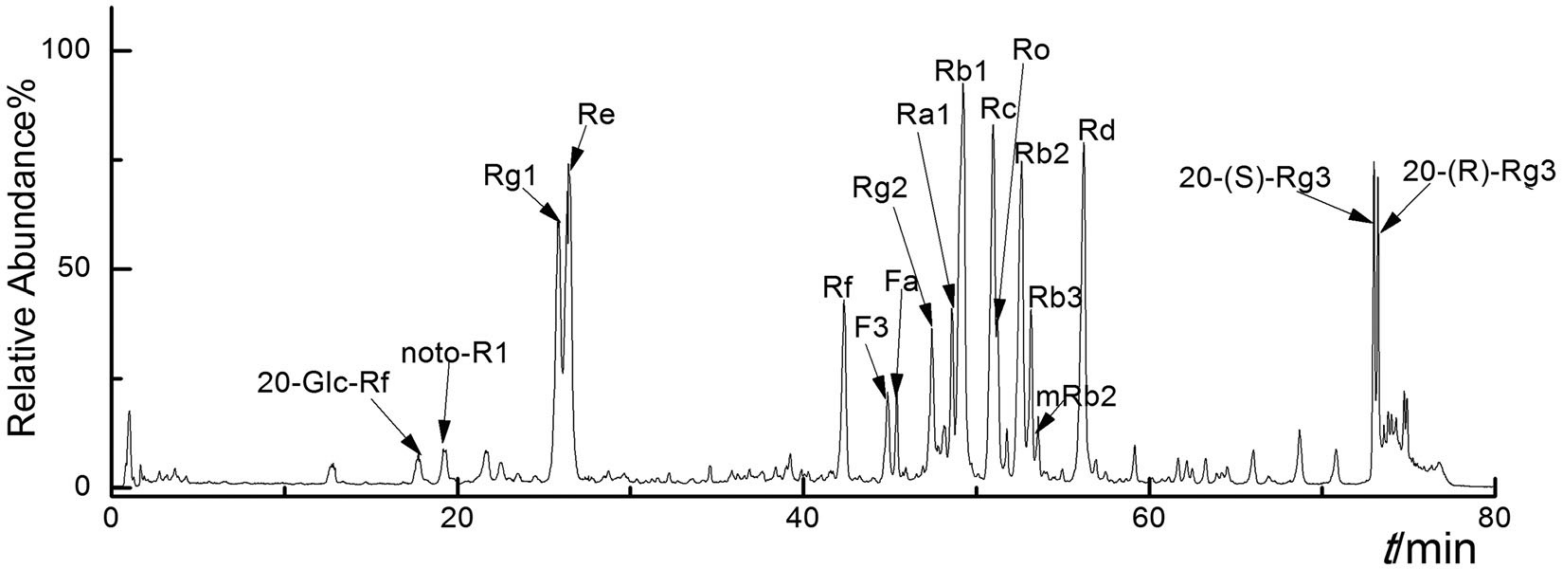
Total ion chromatogram of different ginsenoside monomers in negative ion mode.

According to the fragments, 18 saponin monomers from EEP were identified (Figure 2). Taking Rg1 as reference, the peak areas of the other saponin monomers were taken for the calculation of relative content. To be specific, they were: 20-Glc-Rf (96.154/1077.54, 0.33%), noto-R1 (931.51/977.53, 0.69%), Rg1 (799.48/845.49, 100%), Re (945.54/991.55, 91.96%), Rf (799.48,845.49, 5.58%), F3 (769.47/815.48, 23.26%), Fa (1239.64/1285.64, 5.01%), Rg2 (784.40/829.50, 10.32%), Ra1 (1209.63/1255.63, 13.61%), Rb1 (1107.61/1153.67, 85.84%), Rc (1077.57/1123.39, 50.42%), Ro (955.48, 27.05%), Rb2 (1077.58/1123.59, 67.33%), Rb3 (1077.51/1123.59, 23.45%), mRb2 (1163.58/1209.59, 0.06%), Rd (945.54/991.55, 54.58%), 20-(S)-Rg3 (783.49/829.50, 49.25%) and 20-(R)-Rg3 (783.49/829.50, 38.59%). In EEP, six types of saponin accounted for more than 60% of the content, and they were the main saponin (Rg1, Re, Rb1, Rb2, Rc and Rd) composition in EEP (Figure 2), which may play a major role in its pharmacological activity.

### EEP treatment inhibited weight loss in fatigued rats

Weight gain and changes of diet and water intake were recorded from the time of EEP administration (Table 1). The average weight gain of rats was 60.72 g for the control group and 43.92 g for model group. While the weight gain of the fatigued rats with EEP were 56.67 g (EEP-LG), 49.55 g (EEP-MG) and 53.07 g (EEP-HG). From the point of body weight gain, the fatigued rats with doses of EEP were slightly higher than the rats that without EEP. However, there is no statistical difference (*F*_4,39_=0.578, *p* > 0.05) between the average weights of the groups. In addition, there was also no significant difference in the average diet and water intake between groups.

**Table 1.** Food consumption, water intake and body weight gain.

Groups	Control	Model	EEP-LG	EEP-MG	EEP-HG
Initial weight (g)	248.10 ± 3.91	251.17 ± 6.81	249.91 ± 5.54	250.01 ± 6.69	244.41 ± 3.21
Final weight (g)	308.22 ± 23.20	295.1 ± 25.29	306.58 ± 14.41	300.43 ± 21.01	297.67 ± 26.05
Weight gain (g)	60.72 ± 17.71	43.92 ± 16.03	56.67 ± 18.04	49.55 ± 19.78	53.07 ± 19.34
Food consumption (g/g)	0.058	0.062	0.061	0.064	0.063
Water intake (mL/g)	0.123	0.129	0.122	0.126	0.127

All values were expressed as mean ± SD (*n* = 8/group).

### EEP treatment prolonged swimming time in fatigued rats

**Figure 3.**
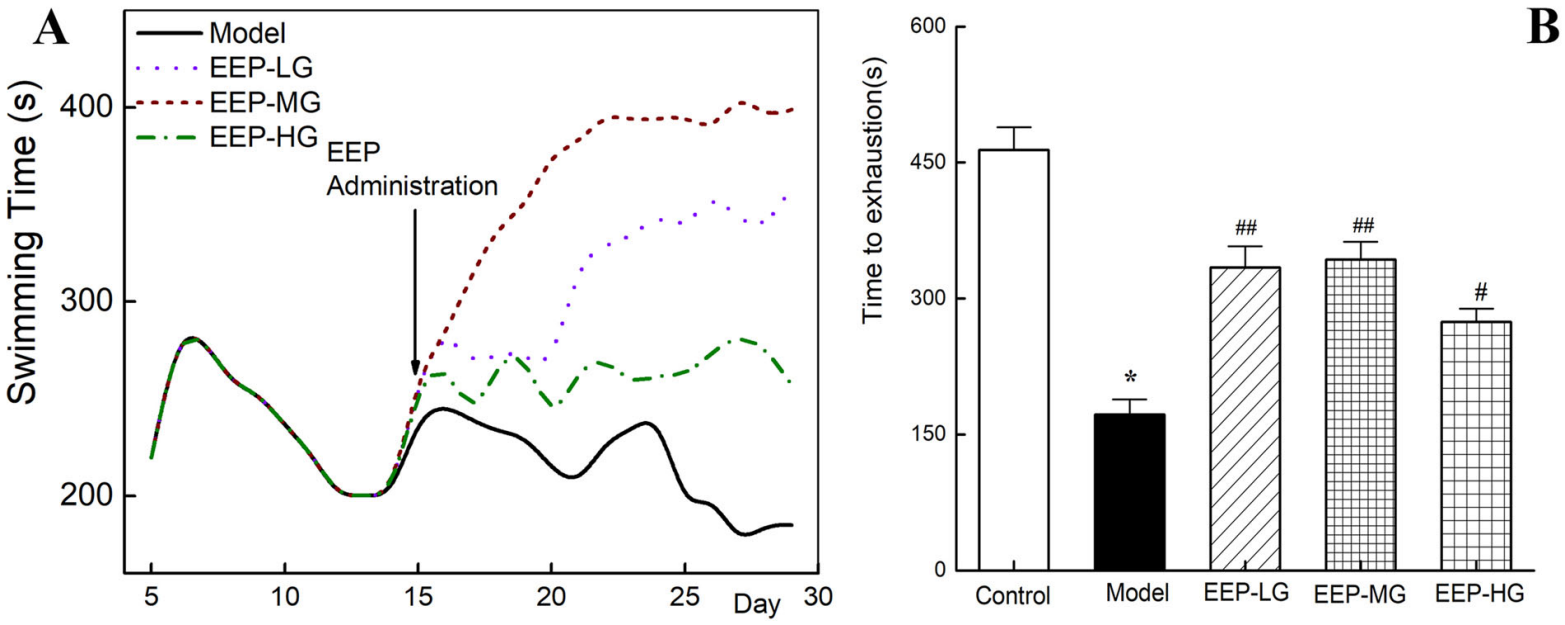
Effects of EEP on loaded swimming time of fatigued rats. (A) Trends of swimming time during the whole swimming period. (B) Swimming times on the last day. *p < 0.05 vs. control group. ^#^*p* < 0.05, ^##^*p* < 0.01 vs. the model group.

As shown in Figure 3, rats’ exhaustive swim time showed a trend of rapid increase (days 5–7) followed by a gradual decrease during the modelling period (days 5–14). On days 12–14, the mean swimming time stabilized, indicating that the animals were in a state of fatigue.

The rats’ swimming time in each group differed as soon as an hour after EEP administration (the 15th day of the experimental schedule). On the 3rd day after administration (the 17th day), the swimming time of the rats in the EEP administration group was significantly (*p* < 0.05) longer than that in the model group. The gap in swimming time between the groups widened further with the duration of EEP administration (Figure 3(A)).

Furthermore, the weight-loaded swimming test was performed again on the 30th day and recorded as presented in Figure 3(B). The exhausting swimming time of the rats in the model group was 172 ± 16.67 s. However, EEP-treated fatigued rats exhibited significantly (*p* < 0.05) better swimming fitness than fatigued rats without any drug intervention, with increases of 94% (EEP-LG), 138% (EEP-MG) and 59% (EEP-HG). This finding indicated that EEP could improve physical performance within an hour. In addition, of the three doses tested, 100 mg/kg resulted in the most significant improvement in physical performance.

### EEP treatment saved the utilization of glycogen, promoted the oxidation of lipid

**Figure 4.**
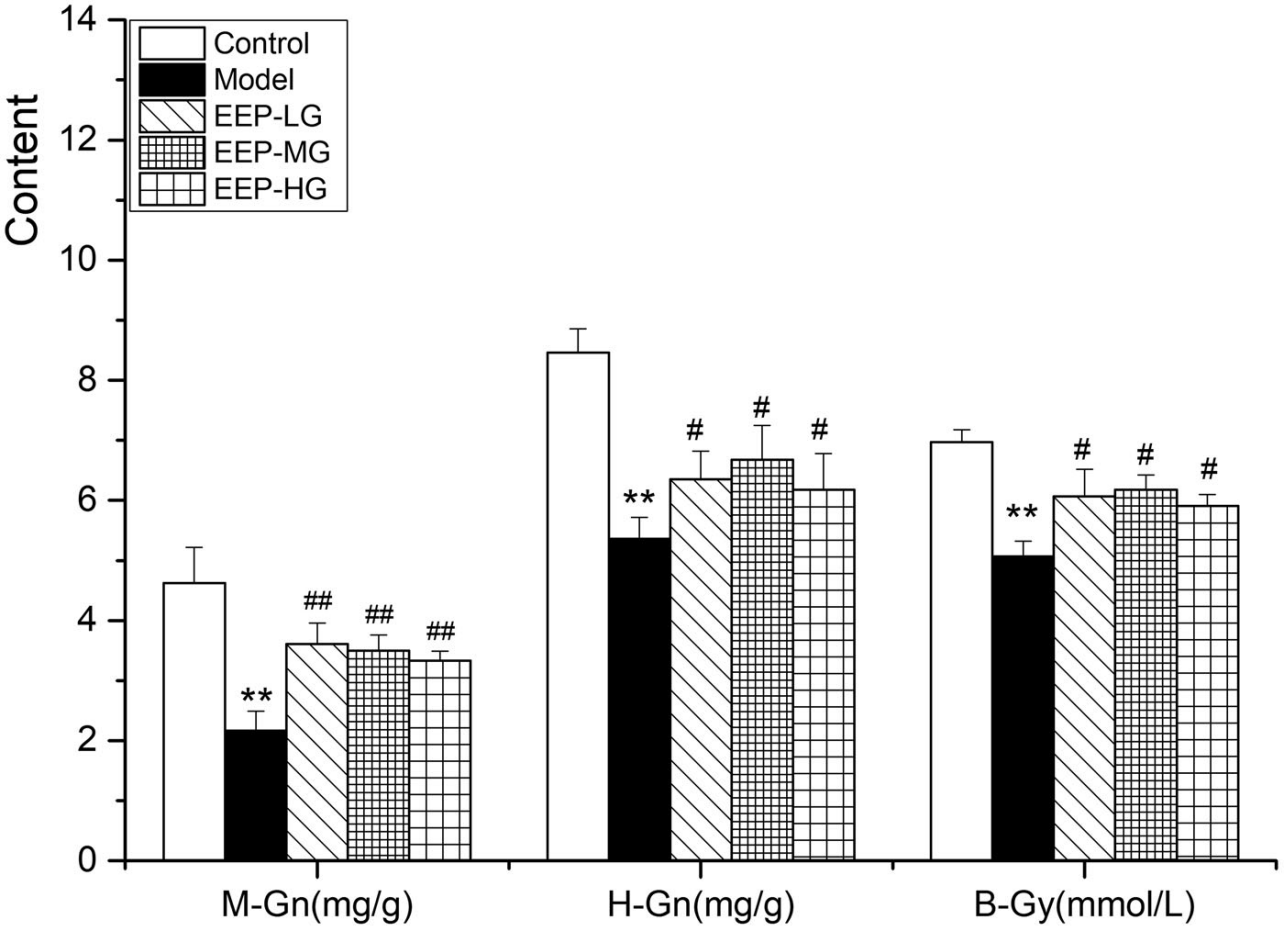
Effects of EEP on M-Gn, H-Gn and B-Gy in fatigued rats. The data are means ± SD (*n* = 8). **p < 0.01 vs. control alone. ^#^*p* < 0.05, ^##^*p* < 0.01 vs. the model.

As shown in Figure 4, M-Gn, H-Gn and B-Gy, levels in fatigued rats treated with doses of EEP significantly (*p* < 0.05) increased compared to rats without medication. Compared to the three doses of EEP, 100 mg of EEP exhibited the best glycogen and B-Gy sparing effects.

**Figure 5.**
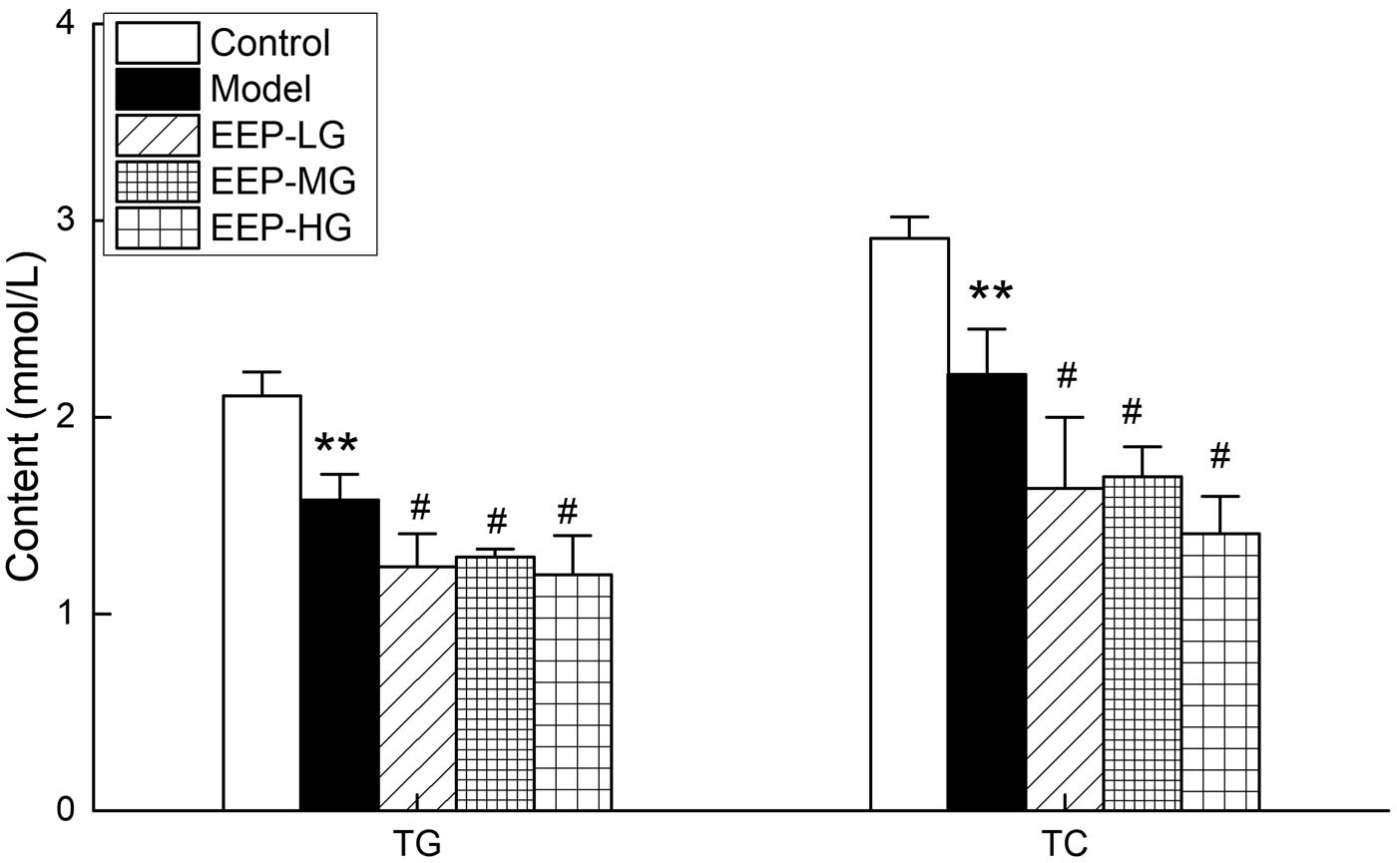
Effects of EEP on blood TG and TC in chronic fatigued rats. Data are shown as means ± SD (*n* = 8). **p < 0.01 vs. control group alone. ^#^*p* < 0.05 vs. the model group.

Figure 5 shows the effect of EEP on TG and TC levels in fatigued rats. The serum levels of TG and TC in model rats were significantly lower than the control group, indicating that chronic fatigue increased TG and TC consumption. EEP significantly reduced the serum TC and TG levels of the fatigued rats compared to the model group.

### EEP treatment reduced LD, BUN and FFA serum levels

**Figure 6.**
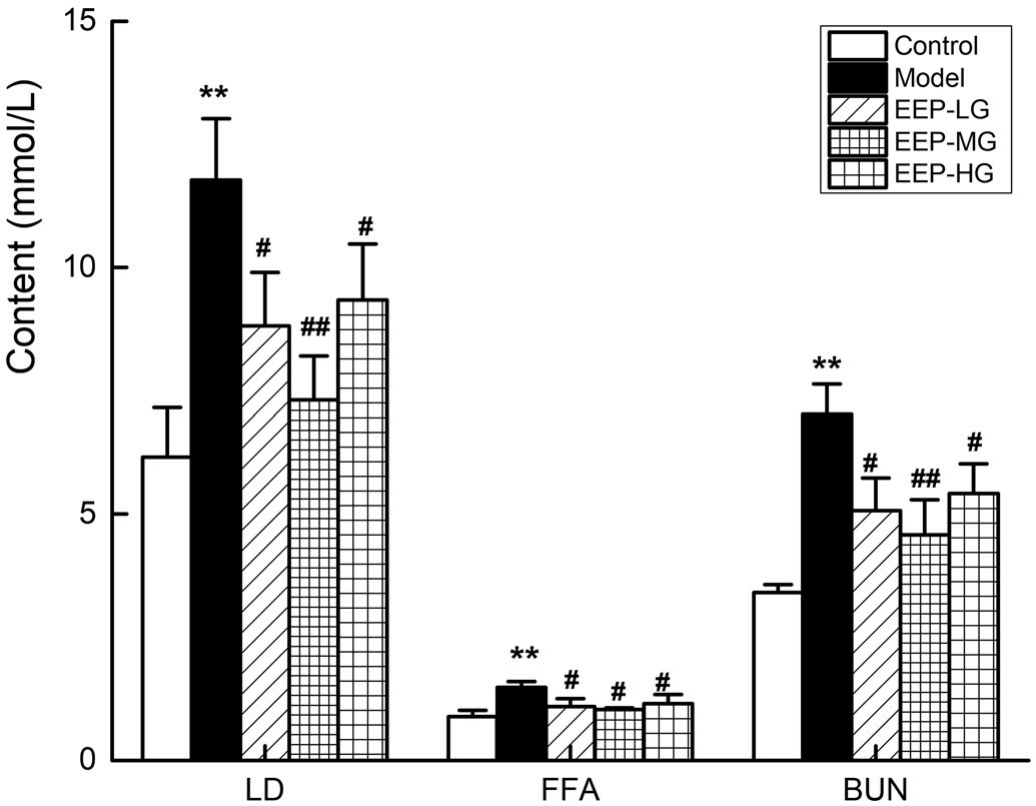
Effects of EEP on serum LD, FFA, BUN in chronic fatigued rats. Data are shown as means ± SD (*n* = 8). **p < 0.01 vs. control group alone. ^#^*p* < 0.05, ^##^*p* < 0.01 vs. the model group.

Serum levels of LD, BUN and FFA are important metabolic indicators of fatigue. After 26 days of weight-loaded swimming training, the LD, BUN and FFA serum levels in the model rats were higher than in the control group (Figure 6). The serum levels of LD, BUN and FFA in fatigued rats receiving different doses of EEP were significantly (*p* < 0.05) lower than in those receiving no medication. Of the three doses, the 100 mg dose was the best. Compared with the model group, the reductions were 38% (LD), 30% (FFA) and 35% (BUN).

### EEP treatment increased Na^+^–K^+^-ATPase and Ca^2+^–Mg^2+^-ATPase activities

**Figure 7.**
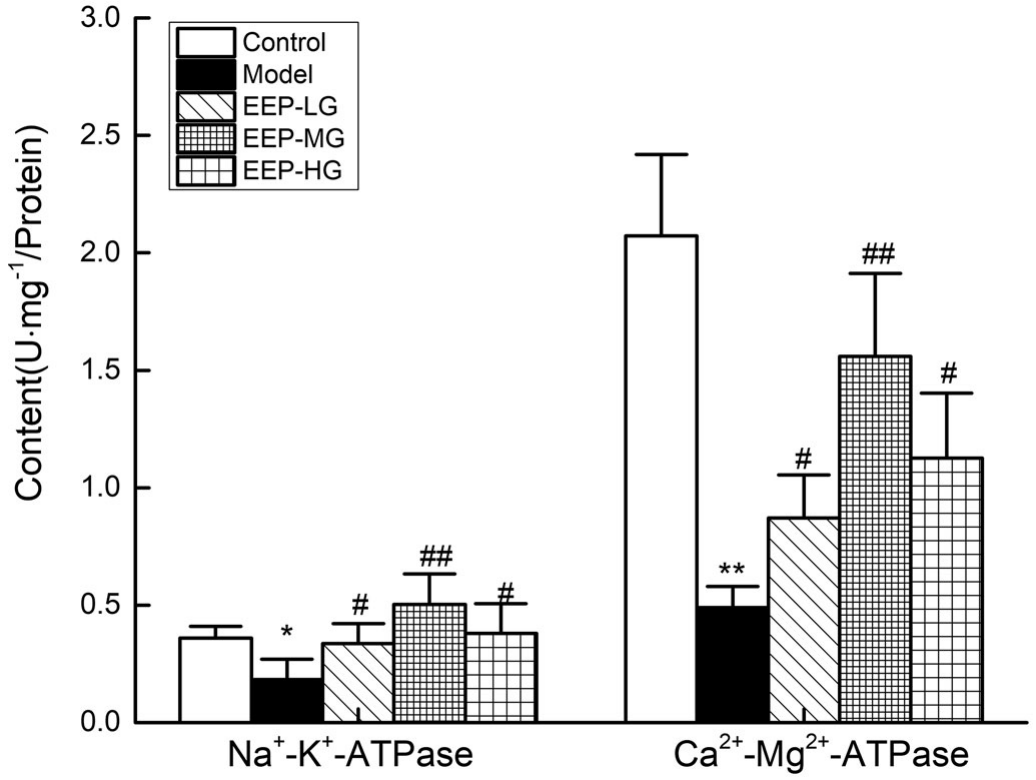
Effects of EEP on Na^+^–K^+^-ATPase and Ca^2+^–Mg^2+^-ATPase in the tissue of skeletal muscle. Data are expressed as the mean ± standard deviation. *p < 0.05, **p < 0.01 vs. control group alone. ^#^*p* < 0.05, ^##^*p* < 0.01 vs. the model group.

The Na^+^–K^+^-ATPase and Ca^2+^–Mg^2+^-ATPase activities are shown in Figure 7. After 26 days of weight-loaded swimming, the Na^+^–K^+^-ATPase and Ca^2+^–Mg^2+^-ATPase activities in rat’s skeletal muscles were significantly (*p* < 0.05) reduced. EEP significantly (*p* < 0.05) increased the Na^+^–K^+^-ATPase and Ca^2+^–Mg^2+^-ATPase activities in rat’ s skeletal muscles than in the model group. The 100 mg/kg dose group exhibited the best promotion of Na^+^–K^+^-ATPase activity, 275% of the model group, which was even better than the control group. When 100 mg/kg dose of EEP was administered to fatigued mice, Na^+^–K^+^-ATPase and Ca^2+^–Mg^2+^-ATPase activities were better than 50 and 200 mg doses.

### EEP treatment mediated the activation of the PI3k/Akt/mTOR pathway

**Figure 8.**
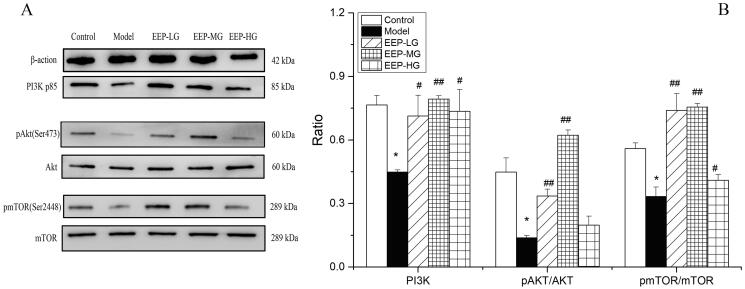
EEP modified the phosphorylation levels of the proteins of the PI3K/Akt/mTOR signal pathway in swimming-induced fatigued rats. (A) The expression levels of PI3K, p-Akt, Akt, p-mTOR and mTOR were detected by western blot analysis. (B) Quantification of PI3K, p-Akt and p-mTOR expression. The data are presented as mean ± SD (*n* = 3). *p < 0.05 vs. control group alone. ^#^*p* < 0.05, ^##^*p* < 0.01 vs. the model group.

Western blot analysis was performed to investigate the modulation of the PI3K/Akt/mTOR signalling pathway on day 30 after EEP administration in rats’ soleus muscle tissues. Specifically, the phosphorylation status of phospho-Akt (Ser 473) and phospho-mTOR (Ser 2448), as an indication of its activation, is mediated by the phosphorylation of the proteins involved. The findings clearly showed a down-regulation of the PI3K/Akt/mTOR pathway in fatigued rats. In detail, a significantly (*p* < 0.05) lower expression of PI3K (p85), p-Akt/Akt and p-mTOR/mTOR (Figure 8(A)) was observed in soleus muscle tissues of fatigued rats compared to the control group. On the contrary, a significant increase in PI3K, p-Akt/Akt and p-mTOR/mTOR was observed in fatigued rats receiving EEP. Among them, an EEP dose of 100 mg/kg/day was associated with the best effect (Figure 8(B)).

## Discussion

The pathological mechanism of fatigue is complex. The current theory on exercise-induced fatigue hypothesizes that it is closely related to energy exhaustion (Wang et al. 2010), accumulation of metabolites, and oxidative stress (Xu and Zhang 2013; Xu et al. 2013). Loaded swimming is a classic method for evaluating fatigue in animals. In previous reports, fatigued mice (rats) were generally swam only once. In this experiment, a chronic fatigue model was used up to 26 days. This study showed that *P. ginseng* could significantly increase the weight-loaded swimming time and improve weight loss in fatigued rats. Specifically, it was demonstrated that the physical strength of fatigued rats was significantly restored with EEP at 50, 100 and 200 mg/kg doses. In addition, six different kinds of saponin monomers (Rg1, Re, Rb1, Rb2, Rc and Rd) accounted for more than 60% of the total content, manifesting that they were responsible for the main antifatigue effect.

In this study, the weight gain of fatigued rats with EEP doses was significantly higher than those without EEP. Most studies did not report the antifatigue effects of drugs on weight gain, while we found that *P. ginseng* inhibited weight loss in rats. The most significant difference is that more than 20 days of weight-loaded swimming training was used in this experiment, much longer than previously reported. Combined with the results reported by Park et al. (2018), it can be concluded that the protective effect of *P. ginseng* on the body changes according to the state of the body; i.e., *P. ginseng* has a bidirectional protective effect on the body, consistent with the report by An et al. (2014). Interestingly, no differences were found in the diet and water intake per gram of body weight of the rats in each group; i.e., EEP did not promote appetite to compensate for energy consumption in fatigued rats.

The exercise-induced physical decline coincides with glycogen consumption, and increasing the storage of liver and muscle glycogen can effectively improve exercise endurance (Gonzalez et al. 2016; Zhao et al. 2018). In this study, *P. ginseng* significantly increased the glycogen reserves of the liver and muscles, which is consistent with the effect of *P. ginseng* reported in the literature (Zhuang et al. 2014). During rigorous exercise, the metabolism and oxidation of carbohydrates and fats cannot meet energy needs. Therefore, protein metabolism is mobilized to maintain the body’s exercise capacity, which produces incomplete oxidation products such as LD, FFA and BUN. The content of BUN is increased; therefore, LD, FFA and BUN levels can reflect the exercise tolerance (Jung et al. 2004). It has been reported that *P. ginseng* can remove or inhibit fatigue metabolites and exert an antifatigue effect (Ma et al. 2017). This study also showed that *P. ginseng* has an antifatigue effect, reduces LA, FFA and BUN levels, and spares glycogen consumption. Na^+^–K^+^-ATPase and Ca^2+^–Mg^2+^-ATPase activities in the process of energy utilization were critical to energy utilization (Boovarahan et al. 2021). Obviously, the activity of Na^+^–K^+^-ATPase and Ca^2+^–Mg^2+^-ATPase in the skeletal muscle of EEP-treated fatigued rats increased dramatically compared to the fatigued rats, consistent with the biochemical indicators.

It is worth mentioning that this study established a fatigue model through 26 days of long-term weight-loaded, exhausting swimming training which was different from the existing antifatigue reports (Tang et al. 2017; Li and Chen 2018). Prolonged fatigue caused massive fat mobilization, resulting in lower TG and TC contents. EEP could further promote the consumption of TG and TC in the body without causing more FFA production, indicating that EEP could make the body’s fat-burning process more complete. It was speculated that this finding might help the application of *P. ginseng* in the auxiliary hypolipidaemic aspect.

In the case of adequate nutrition, ATP synthesis increases with the activation of mTOR. Then, a series of protein substrates are phosphorylated when cells can take in and utilize nutrients to ensure the combination of proteins and fats (Zoncu et al. 2011). In addition, the ATP and glycogen levels objectively reflect energy consumption, while glycogen is the only available substrate for ATP generation when fatigue occurs; thus, the ATP and glycogen levels objectively reflect energy consumption. However, when insufficient ATP synthesis is synthesized in the cell, AMPK will be activated, activating metabolic decomposition processes such as promoting the absorption and metabolism of glucose and fatty acids, inhibiting anabolic processes such as of fatty acids, glycogen, cholesterol and protein. Furthermore, qualitative substances are synthesized to maintain cellular ATP levels (Yang H et al. 2016). As an important upstream factor of the mTOR signalling pathway, PI3K regulates a series of physiological processes and plays an important role in the metabolism of many cells (Abd El Aty et al. 2023). Akt is a downstream signalling molecule of PI3K and an important anti-apoptotic regulator. Together with the adenylate-activated protein kinase (AMP-activated protein kinase, AMPK) signalling pathway, mTOR constitutes cellular anabolic and catabolic processes (Manning and Toker 2017). After Akt is activated, it mainly plays a biological role by promoting the phosphorylation of its downstream substrates. When molecules upstream and downstream of the mTOR pathway are activated by upstream PI3K signalling (such as growth factors) (Craig et al. 2015; Fang et al. 2022) diphosphatidylinositol (PIP2) is converted to triphosphatidylinositol (PIP3) on the cell membrane, further serving as a second messenger to activate downstream proteins. Akt can directly phosphorylate mTOR signalling at Ser2448 to activate mTOR. In this study, up-regulation of phosphorylated mTOR was observed in EEP-administrated rats, indicating that the activation of the PI3K/Akt/mTOR signalling pathway is related to the antifatigue effect of EEP. A similar study was carried out by Fang et al. (2022), who concluded that the Akt/mTOR pathway is a crucial regulator of skeletal muscles’ participation in skeletal muscle hypertrophy, providing energy for muscle cells in mice and preventing muscle atrophy *in vivo*.

Traditional Chinese medicine (TCM) expounds that the spleen is the foundation of acquired constitution, the biochemistry and transportation of Qi, as well as the nourishment of the heart (Li X et al. 2020). Fatigue is a typical manifestation of spleen deficiency. Patients with ‘spleen deficiency’ have nutrient absorption disorders, which can be manifested as diarrhoea, abdominal distension, weight loss, malnutrition, weakness of limbs, and decreased anti-stress ability, etc. These symptoms are like the rat fatigue model established in this experiment. In TCM, *P. ginseng* belongs to the spleen, lung and heart meridian, which could nourish the Qi of the spleen, lungs and heart, enrich the acquired essence and nourish the innate essence. ‘Reinforcing vital energy’ was the classic effect of *P. ginseng* (Sun et al. 2016). This study shows that medicinal and edible *P. ginseng* could quickly restore the physical strength of fatigued rats and prevent weight loss. *P. ginseng* can improve the sugar uptake ability and glycogen synthesis ability of skeletal muscle cells in fatigued rats, reduce blood LD value and serum urea nitrogen content, prolong the swimming time of rats, and finally show an anti-fatigue effect. This result is consistent with the traditional effect of *P. ginseng* in TCM.

## Conclusions

This study showed that *P. ginseng*, one of the most popular Chinese tonic herbs, is a promising sports ergogenic aid. *P. ginseng* triggers metabolic adaptation to energy expenditure by activating the PI3K/Akt/mTOR signalling pathway, sparing the utilization of glycogen and protein during exercise by promoting the oxidative energy supply of fat and reducing LD, BUN and FFA serum levels. Short-term EEP supplementation resulted in a significant increase in exercise capacity. Overall, *P. ginseng* has a good antifatigue effect. Our finding suggests that EEP effectively relieves fatigue, providing evidence for its potential as a therapy herb against fatigue.
